# Management of genetic erosion: The (successful) case study of the pear (*Pyrus communis* L.) germplasm of the Lazio region (Italy)

**DOI:** 10.3389/fpls.2022.1099420

**Published:** 2023-01-09

**Authors:** Samela Draga, Fabio Palumbo, Immacolata Miracolo Barbagiovanni, Francesco Pati, Gianni Barcaccia

**Affiliations:** ^1^ Department of Agronomy, Food, Natural Resources, Animals and the Environment, University of Padova, Padua, Italy; ^2^ ARSIAL, Agenzia Regionale per lo Sviluppo e l'Innovazione dell'Agricoltura del Lazio, Via Rodolfo Lanciani, Roma, Italy

**Keywords:** pear germplasm, genetic resources, molecular characterization, variety maintenance, conservation, regional registers

## Abstract

*Pyrus communis* L. is an important temperate fruit with high nutritional and economic value. Italy, as the largest pear producer in the EU and second in the world, has a particularly rich germplasm characterized by hundreds of local varieties. The Lazio Region was the first Italian region to start programs focused on safeguarding varieties at risk of extinction and has started a massive census of fruit varieties by combining molecular data and productive-territorial information. In this study, 311 pear accessions collected from the five provinces of the Lazio region were genetically characterized by the means of nine simple sequence repeat (SSR) markers, eight of which were recommended by the European Cooperative Programme for Plant Genetic Resources (ECPGR). The SSR analysis revealed 250 unique genotypes and 14 cases of synonymies, namely, accessions with different names but identical molecular profiles (100% genetic similarity). The microsatellite set showed a high polymorphism information content (PIC; mean PIC=0.77) and an exceptionally high discrimination power (DP = 0.99), making it particularly efficient both for the study of genetic diversity and for the prediction of the degree of ploidy. Notably, 20% of the accessions displayed triallelic profiles (i.e., accessions having ≥2 loci with a third allele), while the remaining accessions were diploids. These results were further confirmed by flow cytometry data analysis. Standardization of the molecular analyses at the national and international levels and harmonization of the SSR sets used for germplasm characterization are of paramount importance for producing molecular profiles useful for registration and variety maintenance.

## 1 Introduction

*Pyrus communis* L. is an ancient fruit belonging to the *Pyrus* genus in the family Rosaceae that is widely known for enriching our dietary intake. The genus has a basic number of chromosomes (x = 17), and the cultivated forms are mainly diploid and triploid (Darlington and Moffett, 1930; Sehic et al., 2012; Urrestarazu et al., 2015; Baccichet et al., 2020).

The genus originated in the mountainous regions of southwestern China and is one of the oldest fruit crops in the world, with a long history of cultivation of more than 3,000 years (Simmonds, 1993; Hancock and Lobos, 2008). Its vast distribution is due to its wide commercial appreciation around the world, its nutritional importance, and its adaptability in places with wide planting conditions and marketing (Silva et al., 2014). The exploration of the pear germplasm in the Italian Peninsula dates from the period of the Roman Empire. Among the Roman historians, Pliny the Elder made a great contribution, describing in detail all the varieties of the season in a manuscript with more than sixty editions (Janick, 2002; Eccher and Pontiroli, 2005). Moreover, the ancient Romans reported that more than 40 cultivars existed in the 1^st^ century BC and described methods of cultivation comparable to those currently practiced (Miranda et al., 2010; Saito, 2016). The history of the pear has traced from antiquity to the present, in which Italy is a top producer in the world, ranking second after China (FAO STAT, 2020). Beyond the commercial production of improved varieties in specialized and extensive orchards, Italy has a wealth of local and traditionally-managed pear orchards that have the potential to provide unique habitats for wildlife and that tend to hold older and rarer varieties of fruit. However, they are constantly threatened, due to their progressive replacement with high yielding and more uniform commercial varieties (Ferradini et al., 2017).

In Italy, each region has a public institution that coordinates the major actions of conservation and valorization of biodiversity due to their deep local knowledge and the legislative autonomy in agriculture. In the early 2000s, the Lazio Region, through the Regional Agency for the Development and Innovation of Lazio Agriculture (ARSIAL), was the first Italian region (and among the first in Europe) to start programs focused on safeguarding local varieties at risk of extinction (Regione Lazio, 2000). Initially, the Regional Council established specific criteria to define the degree of risk of genetic erosion of a local variety and to register these varieties in a special repository to protect their genetic patrimony (Programma di Sviluppo Rurale Lazio, 2013). Among them, we find the number and age of farmers cultivating a specific variety, the cultivated surface in relation to the total regional agricultural area, the distribution of the cultivated area, the type of market, trends in new systems and cultivations and the presence of activities for ex situ conservation. However, since the genetic erosion of a variety consists of the progressive restriction of its gene pool, in recent years, there has been a growing debate about the need to integrate the information provided by the abovementioned descriptions with molecular data encompassing the genetic background of a variety. The use of molecular analysis allows i) estimation of the relationships existing between individuals to separate them into groups according to geographical areas, ii) discrimination of accessions that are phenotypically very similar, iii) identification of a variety through unique molecular profiles, and iv) detection of duplicate accessions (especially in the constitution of *in situ* and ex situ collections) (Pereira-Lorenzo et al., 2012; Baccichet et al., 2020; Montanari et al., 2020; Bielsa et al., 2021). Furthermore, the use of particular classes of codominant molecular markers, such as SNPs (single nucleotide polymorphisms) and SSRs (simple sequence repeats), supports the standardization of the analysis method, increasing the repeatability and comparability of the results (Evans et al., 2007). This is of pivotal importance for the continuous monitoring of the gene pool of a variety over time. Finally, it should not be forgotten that the genetic characterization of local varieties provides the basis for the construction of core collections and the designation of genetically unique accessions, enhancing their use in breeding programs (Tatari et al., 2020; Bielsa et al., 2021). In this context, the Lazio region has started a massive census of fruit and vegetable varieties by combining molecular data with productive-territorial information. The success of this approach has convinced both other Italian regions and the government itself (through the Ministry of Agriculture) to pursue similar measures (Ministero delle politiche agricole alimentari e forestali, 2015).

In this study, assisted by ARSIAL, CREA-OFA (The Council for Agricultural Research and Analysis of Agricultural Economics - Olive, Fruit and Citrus Crops Center), local farmers and horticulturists, we molecularly characterized the pear germplasm of the Lazio region, collecting 313 accessions from all five provinces of the region. Among the main objectives were i) determining the genetic identity of the pear accessions, ii) elucidating cases of homonymy and synonymy, and iii) producing molecular profiles useful for the registration and safeguarding of local varieties.

## 2 Materials and methods

### 2.1 Plant material, DNA extraction and amplification

In total, 313 accessions collected from all five provinces of the Lazio region were provided by the CREA-OFA center and by local farmers and horticulturists. The collection consisted of 104 samples from Frosinone, 66 from Latina, 69 from Rieti, 57 from Rome and 15 from Viterbo. For each accession, one representative sample was collected, considering that pear trees are almost exclusively propagated by grafting (i.e., vegetative propagation). Genomic DNA samples (gDNA) were extracted from young leaves using the DNeasy Plant Mini Kit (Qiagen, Hilden, Germany) according to the manufacturer’s protocol. After extraction, the gDNA quality and quantity were evaluated using a NanoDrop 2000c UV−Vis spectrophotometer (Thermo Fisher, Pittsburgh, PA, United States). The DNA sample integrity was checked by electrophoresis on a 2% agarose/1× TAE gel containing 1× Sybr Safe DNA gel stain (Life Technologies). Twelve SSR markers were chosen from the literature (Liebhard et al., 2002; Hemmat et al., 2003; Fernández-Fernández et al., 2006; Gasi et al., 2013) based on their polymorphism information content (PIC) and sequence length (**Table 1**). The forward primer of each couple was labeled with a 20 bp oligo tail, complementary to a third primer (i.e., M13, PAN1, PAN2, and PAN3) labeled, respectively, with a fluorescent molecule (6-FAM, VIC, NED and PET). This three-primer system was originally described by Schuelke et al. (Schuelke, 2000) and modified by Palumbo et al. (Palumbo and Barcaccia (2018)). Tests on the amplification efficiency of each SSR locus were conducted on a subset of six accessions. Nine (out of 12) SSR marker loci were then selected and organized into two multiplexes based on the primer annealing temperature, amplicon size, amplification efficiency, and dimer formation tendency (**Table 1**, in bold). Finally, the two multiplexes were used to analyze the entire germplasm.

**Table 1:** List of the selected primer pairs.

Locus	Forward primer	Reverse primer	Size (bp)	Tail	Ta (°C)	MP	Ref.
**GD147**	TCCCGCCATTTCTCTGC	AAACCGCTGCTGCTGAAC	134	M13	57	1	(Hemmat et al., 2003)
**CH01f07a**	CCCTACACAGTTTCTCAACCC	CGTTTTTGGAGCGTAGGAAC	190	M13	57	1	(Hemmat et al., 2003)
CH02c02a	CTTCAAGTTCAGCATCAAGACAA	TAGGGCACACTTGCTGGTC	152	PAN1	57	1	(Liebhard et al., 2002)
**EMPc11**	GCGATTAAAGATCAATAAACCCATA	AAGCAGCTGGTTGGTGAAAT	141	PAN2	57	1	(Fernández-Fernández et al., 2006)
CH02c11	TGAAGGCAATCACTCTGTG	TTCCGAGAATCCTCTTCGAC	229	PAN2	57	1	(Liebhard et al., 2002)
**GD142**	GGCACCCAAGCCCCTAA	GGAACCTACGACAGCAAAGTTACA	163	PAN3	57	1	(Gasi et al., 2013)
**CH01d03**	CCACTTGGCAATGACTCCTC	ACCTTACCGCCAATGTGAAG	150.	M13	60	2	(Liebhard et al., 2002)
**CH02b10**	CAAGGAAATCATCAAAGATTCAAG	CAAGTGGCTTCGGATAGTTG	140	PAN1	60	2	(Liebhard et al., 2002)
**CH04e03**	TTGAAGATGTTTGGCTGTGC	TGCATGTCTGTCTCCTCCAT	200	PAN1	60	2	(Liebhard et al., 2002)
**CH01d09**	GCCATCTGAACAGAATGTGC	CCCTTCATTCACATTTCCAG	153	PAN2	60	2	(Liebhard et al., 2002)
CH03g07	AATAAGCATTCAAAGCAATCCG	TTTTTCCAAATCGAGTTTCGTT	150	PAN3	60	2	(Liebhard et al., 2002)
**CH01d08**	CTCCGCCGCTATAACACTTC	TACTCTGGAGGGTATGTCAAAG1	264	PAN3	60	2	(Liebhard et al., 2002)

The SSR locus name, forward and reverse sequences, expected fragment sizes (bp), anchor, annealing temperature, and multiplex (MP) are reported. Those loci that were retained after preliminary analyses and used to analyze the entire germplasm are highlighted in bold.

The PCR was carried out in a final volume of 20 µL containing 10 µl of 2X Platinum (Thermo Scientific, Carlsbad, CA, USA), 0.5 µM of each tailed forward primer, 0.75 µM of each reverse primer, 0.25 µM florescent primer mix (Applied Biosystems, Carlsbad, CA, USA), 40 ng of gDNA, 1 µl of GC Enhancer (Thermo Scientific), and sterile distilled H_2_0 up to a final volume of 20 µL. Amplification was performed in 96-well plates using a 9700 Thermal cycler (Applied Biosystems, Foster City, CA, USA) under the following thermal conditions: 5 min at 95°C, followed by 40 cycles at 95°C for 30 s, 60°C for 45 s, and 72°C for 45 s. Reactions were terminated with a final extension of 30 min at 60°C. Ten nanograms of each PCR product was subjected to capillary electrophoresis on an ABI PRISM 3130xl Genetic Analyzer (Thermo Fisher) using LIZ500 (Applied Biosystems) as the molecular weight standard and G5 as a filter.

### 2.2 Data analysis

After capillary electrophoresis, the size of each peak was manually determined using Peak Scanner v1.0 software (Applied Biosystems). This allowed us both to estimate the ploidy of each accession and to create a dataset of alleles for 311 pear accessions. Two samples were excluded due to missing data.

The discrimination power (DP) of the SSR set was calculated as defined by Tessier et al. (1999):


$$DP=1-\sum_{n=1}^{I}p_{i}²$$

where *p_i_* represents the frequency of the **i**-th genotype and **I** represents all the genotypes (311) analyzed in this study.

All the other SSR statistics were calculated using POPGENE v. 1.32 software (Yeh et al., 1997) by excluding triploid genotypes. For the statistics, we estimated the observed (Ho) and expected (He) heterozygosity, the average (na) and the effective (ne) number of alleles per locus, and the Shannon index (I). To determine the informativeness of the assessed marker loci, Nei’s (Nei, 1973) index was calculated and assumed to represent the polymorphic index content (PIC).

### 2.3 Genetic similarity and genetic structure analyses of the core collection

Genetic similarity (GS) estimates were calculated between individuals in all pairwise comparisons by applying the simple matching coefficient using NTSYS-pc v. 2.21q software (Rohlf, 1988). The resulting triangular similarity matrix (311 x 311) was used for the construction of a principal coordinate analysis (PCoA).

Analysis of molecular variance (AMOVA) was computed, grouping the accessions according to geographic location. GenoDive v6.5 software (Peakall and Smouse, 2012) was used to perform the AMOVA and to analyze the molecular variance at different levels of population structure with 999 permutations.

The genetic structure analysis was employed using the Bayesian clustering algorithm implemented in STRUCTURE v2.2 software (Porras-Hurtado et al., 2013) to infer the most likely number of K with a burning period of 2·10^5^ and a final run of 10^6^ MCMC (Monte Carlo Markov Chain) replicates. The optimum number of populations (K) was calculated using STRUCTURE harvester web-software as described by Evanno et al. (Evanno et al., 2005). The obtained results were plotted as histograms with a vertical bar for each accession divided into K colored segments and used to represent the estimated membership in each hypothesized ancestral genotype, and the estimates of membership were plotted as a histogram using an Excel file.

Genetic relationship analysis was performed according to the maximum likelihood method (ML) implemented in IQ-Tree v1.6.12 software (Nguyen et al., 2015). The matrix resulting from the SSR marker dataset (311 samples) was analyzed as binary data using the GTR2 method (GTR2+I+G4+FO), according to the BIC value determined with the ModelFinder algorithm available in IQ-Tree. The GTR model, used to investigate the genetic relationship with SSR data, was selected according to scientific research (Vieira et al., 2016). Statistical support for the ML dendrogram was computed by running 1000 replicates until convergence for ultrafast bootstrap (UFB) (-bb 1000) (Guindon et al., 2010; Thi Hoang et al., 2017).

### 2.4 Ploidy level and genome size estimate by flow cytometry

To confirm the ploidy level predicted through the SSR analysis, 34 putative diploids and 16 putative triploids randomly selected within the germplasm were further investigated through flow cytometry (CyFlow Ploidy Analyzer, Sysmex, DE) by means of 4′,6-diamidino-2-phenylindole (DAPI)-stained nuclei, following the procedure described by the CyStain UV Precise protocol. Approximately 0.5 cm^2^ of young fresh leaves were chopped with a razor blade in a Petri dish with 0.5 ml of Nuclei Extraction Buffer (Sysmex Partec) and incubated for 5 minutes at room temperature. After filtering (30 μm CellTrics^®^, Sysmex, DE), 2 ml of staining buffer was added to each sample and incubated for 60 s before analysis (Nd-YAG green laser: λ = 532 nm; 30 mW, flow rate of 4 μl/s). Fluorescence histograms were evaluated using FCS Express 5 Flow software (Sysmex).

The genome size of three diploids (Invernale FR, Spina 2 FR, Di Carpello RM) and three triploids (Cocozzola FR, Bottiglia FR, Sammonatana FR) was determined through flow cytometry of propidium iodide (PI)-stained nuclei, following the procedure described by the CyStain PI Absolute P protocol (Sysmex 107 Partec, Görlitz, Germany). *Raphanus sativus* and *Solanum lycopersicum* seeds with known 2C DNA content were kindly provided by Prof. Dolezel (https://olomouc.ueb.cas.cz/en/technology/flow-cytometry-1/reference-dna-standards) and adopted as reference standards. The analysis was conducted by cochopping each of the six samples with both references. Approximately 0.5 cm^2^ of young leaves for each accession were chopped with a razor blade along with 0.5 ml of Nuclei Extraction Buffer (Sysmex Partec), incubated for 5 minutes at room temperature and filtered using 30 μm CellTrics (Sysmex Partec). Two milliliters of staining solution (1820 μl of Staining Buffer, 120 μl of PI and 60 μl of RNAse A 3.3 ng/μl) was then added to each filtered sample, and the resulting solution was placed on ice in the dark for 5 minutes. Analyses were run using the following parameters: Nd-YAG green laser: λ = 532 nm; 30 mW, flow rate of 2 μl/s. Fluorescence histograms were evaluated using FCS Express 5 Flow software (Sysmex Partec), and *c* values were inferred by comparing the sample and standard at G0/G1 peak positions.

## 3 Results and discussion

Microsatellite markers or SSRs have been widely used in the last 20 years for a wide range of purposes, including genetic diversity analysis, parentage assessment and genetic map development (Feng et al., 2016). Although SSRs are giving way to newer genotyping technologies, such as restriction site-associated DNA sequencing (RAD-seq) and genotyping by sequencing (GBS), they remain valuable tools for three main reasons. Newer technologies guarantee the identification of a number of markers per sample far greater than that identifiable with SSR markers (a few thousand vs. a few tens, respectively) but have a significantly higher cost per sample. Thus, in conservation genetic studies, where priority is not represented by a high marker density per sample but rather by the inclusion of a large number of samples, the use of SSR markers still represents a reasonable alternative. Moreover, having been used for several years and in thousands of studies, the use of SSR benefits from the presence of enormous databases and a very in-depth literature. Finally, and most importantly, in conservation genetic surveys (including the present study) that are often funded with a very low budget, SSRs continue to be the most economical option (Jennings et al., 2011). In fact, in our study, we demonstrated that a small but highly polymorphic set of SSRs remains a good choice for the characterization of entire germplasms threatened by genetic erosion.

### 3.1 SSR statistics and comparison with previous studies

After a preliminary analysis with 12 SSR marker loci on a subset of six samples, 9 SSR markers were selected and arranged in two multiplex reactions (**Table 1**, in bold). The remaining three loci (CH02c02a, CH02c11, and CH03g07) were excluded from the study because of poor amplification and unreliable SSR profiles. One hundred forty-four different alleles were scored from the 9 SSR markers (on average 16 per locus), ranging from 9 (CH04e03) to 23 (GD142, **Table 2**).

**Table 2:** SSR descriptive statistics reporting the sample size and the number of observed alleles (Na) of all 311 individuals successfully amplified for each locus.

Marker	Sample Size	Na	Ho	He	I	PIC
GD147	311	12	0.68	0.74	1.67	0.73
CH01f07a	311	18	0.71	0.82	2.02	0.82
EMPc11	310	11	0.80	0.79	1.83	0.78
GD142	302	23	0.88	0.87	2.33	0.86
CH01d03	310	19	0.92	0.90	2.42	0.90
CH02b10	300	17	0.49	0.84	2.07	0.84
CH04e03	311	9	0.27	0.28	0.72	0.29
CH01d09	307	20	0.89	0.91	2.53	0.91
CH01d08	307	15	0.78	0.81	1.84	0.81
**Mean**	**308**	**16**	**0.71**	**0.77**	**1.94**	**0.77**
St. Dev.	4.1	4.6	0.21	0.19	0.54	0.19

Observed heterozygosity (Ho), expected heterozygosity (He), Shannon’s information index (I) and polymorphic informationcontent (PIC) were calculated only for diploid genotypes (n=161).

As expected, this exceptional allelic richness also resulted in very high polymorphism information content (PIC) coefficients. PIC values ranged from 0.29 (CH04e03) to 0.91 (Ch01d09), with a mean value of 0.77 (**Table 2**). According to Botstein et al. (Botstein et al., 1980), all the SSRs adopted in this analysis proved to be highly informative (PIC values were always higher than 0.73) with only the exception of CH04e03.

Another useful parameter to evaluate the informativeness of a marker set is the probability of identity (PI), namely, the probability that two individuals shared the same genotype by chance and not by kinship. The lower the PI value is, the greater the power of discrimination (DP) value. The power of discrimination (DP) of the nine SSR markers calculated in 311 samples was very high (DP = 0.99) as a further confirmation of their efficiency.

Therefore, due to its robustness and reliability, the use of this SSR set is highly recommended for future studies aimed at an accurate genetic identification and diversity assessment of pear germplasm.

One of the main issues related to the use of molecular markers for varietal identification consists of the difficulty encountered in standardizing the molecular analyses at the national and international levels. Harmonization of the SSR sets used for germplasm characterization is pivotal to compare data obtained in different studies. An agreement has been reached only for a few major species, such as grapevine and apple, mainly due to the coordinating role played at the international level by the OIV (International Organisation of Vine and Wine) and the ECPGR (European Cooperative Programme for Plant Genetic Resources), respectively. As a result, the international databases for the molecular registration of grapevine and apple varieties rely on two gold standard sets of markers, consisting of 9 (Lateur et al., 2013) and 12 (OIV, 2019) SSRs, respectively. An attempt to standardize the method in *Pyrus* was made by the ECPGR through the proposal of a 12 SSR set (Evans et al., 2007). Although some authors pointed out the difficulties encountered in the use of this set, due to the amplification of artifacts (stutters and split peaks) (Evans et al., 2015; Zurn et al., 2020), in our study, we tried to strictly adhere to the proposal. In fact, 8 out of 9 SSRs were among those proposed by the ECPGR. An additional locus (CH01d03) was chosen due to its high PIC value. However, by performing a meta-analysis of the main European studies conducted in the last 20 years in pear, we found that only a few studies (dos Santos et al., 2011; Urrestarazu et al., 2015; Bielsa et al., 2021; Velázquez-Barrera et al., 2022) rigorously followed the proposed SSR list (**Table 3**). Among the SSR loci, the most frequently adopted were CH01d09, CH01f07a, EMPc11, CH04e03 and CH02b10. This tendency to ignore the guidelines makes it very challenging to compare the results obtained from different studies and to create common databases. Furthermore, the fact that only a few studies reported PIC values makes it difficult to define the true informativeness of each locus. For example, from the limited data available, it was possible to ascertain how the least informative SSR in this study (CH04e03 locus, PIC = 0.29) also resulted in the least informative locus in all the other studies for which the PIC was available (range 0.34-0.55, **Table 3**). There is still a long way to go before an international agreement on the use of a reference set in *Pyrus* is reached.

**Table 3:** Comparison of the research studies conducted in the last 20 years in Europe on *Pyrus*.

Country/Organization	Author	No. of samples	GD147	CH01f07a	EMPc11	GD142	CH01d03	CH02b10	CH04e03	CH01d09	CH01d08
Italy	Present study	311	0.73	0.82	0.78	0.86	0.9	0.84	0.29	0.91	0.81
ECPGR	(Evans et al., 2007)	–	•	•	•	•		•	•	•	•
Belarus	(Urbanovich et al., 2011)	43								0.83	0.85
Bosnia and Herzegovina	(Kajkut Zeljković et al., 2021)	74	0.65	0.85	0.85				0.55	0.9	0.76
Bosnia and Herzegovina	(Gasi et al., 2013)	64		•	•		•	•	•	•	
Bosnia and Herzegovina	(Salkić et al., 2021)	9		•	•		•	•	•	•	
Germany	(Reim et al., 2017)	278	•	•				•		•	
Germany, Romania	(Puskás et al., 2016)	188	0.39	0.77	0.58			0.75	0.42	0.67	0.54
Hungary, Austria	(Kocsisné et al., 2020)	81	•						•	•	
Italy	(Baccichet et al., 2020)	170	0.68	0.87	0.80			0.85	0.50	0.91	0.75
Italy	(Sau et al., 2020)	108	•	•	•			•	•	•	
Italy	(Bennici et al., 2018)	95							0.42		
Lithuania	(Rugienius et al., 2013)	84			•		•				
Lithuania	(Lukoseviciute et al., 2013)	45			•						
Norway	(Meland et al., 2018)	8		•	•		•	•	•	•	•
Portugal	(Queiroz et al., 2019)	130		0.65	0.55			0.74	0.34	0.73	0.66
Portugal	(Queiroz et al., 2015)	54		0.88	0.84						0.69
Portugal	(Bassil et al., 2009)	7		•				•		•	•
Spain	(Velázquez-Barrera et al., 2022)	266	•	•	•	•	•	•	•	•	•
Spain	(Bielsa et al., 2021)	252	•	•	•	•		•	•	•	•
Spain	(Urrestarazu et al., 2015)	244	•	•	•	•	•	•	•	•	•
Spain	(dos Santos et al., 2011)	241	•	•	•	•	•	•	•	•	•
Spain	(Segura et al., 2021)	170	•	•	•	•		•	•	•	•
Spain	(Fernández‐Fernández et al., 2006)	21		•							
Sweden	(Sehic et al., 2012)	94	•	•	•				•	•	•
**Average**		127	0.61	0.83	0.75	0.86	0.90	0.81	0.44	0.85	0.72

For each study, we reported the country of origin, references, number of samples analyzed and SSRs in common with the present study. PIC values were also reported (if available).

As for other SSR statistics, both the mean values of observed and expected heterozygosity among all accessions were very high (0.71 and 0.77, respectively, **Table 2**) and comparable with results reported for pear genotyping (Darlington and Moffett, 1930; Sehic et al., 2012; Urrestarazu et al., 2015; Bielsa et al., 2021). These values were also consistent with the allogamous nature of the species, mainly because of an efficient gametophytic self-incompatibility (GSI) system that promotes outbreeding and prevents self-fertilization (Bennici et al., 2020).

### 3.2 Synonymy and homonymy cases within the pear germplasm

One of the main complications in germplasm management arises from the confusion accumulated over the centuries in the assignment of varietal names, leading to numerous cases of synonymy (one genotype with several denominations) and homonymy (one denomination for several genotypes) (Muzzalupo et al., 2014).

Overall, the SSR set employed in this study allowed the identification of 250 different allelic combinations, hereafter defined as genotypes. The fact that the number of genotypes (250) was lower than the number of accessions analyzed (311) demonstrated the presence of accessions sharing the same genotype and therefore cases of synonymy. Supported by the genetic similarity matrix (**Supplementary Table 1** **)**, we identified 26 cases in which two or more accessions shared the same genotype. In some cases, they shared very similar or identical names (e.g., ‘Abitir’ collected in Latina and ‘Abitir’ collected in Rieti). In contrast, in 14 cases (**Supplementary Table 2**), accessions with identical genotypes did not share similar varietal names. From the literature, it seems that some varieties assume different names according to the province of origin. For instance, this is the case for ‘Capattera’ (Frosinone) and ‘Campanella’ (Latina), which, in fact, shared the same genotype. Similarly, the variety ‘Curato’ is internationally recognized by different names, and while in this study it was synonymous with ‘Bottiglia’, in other regions from northern and central Italy, it is considered synonymous with ‘Spada’ or ‘Spadona’ (Bianco et al., 2014; Baccichet et al., 2020).

In rare situations, synonymy can be the result of mislabeling practices, especially because of high phenotypic similarity, such as for the ‘Al Burro’ and ‘Fegatella’ accessions. Since they are both harvested in September-October and are characterized by a peculiar very dark skin, we cannot exclude that the one has been confused with the other.

Regarding the homonymy cases, it was evident how varieties that were demonstrated to be discriminable from a molecular point of view have been identified for a long time with the same name, probably due to a strong phenotypic similarity. Of note is the case of the ‘Spina’ variety. Nineteen accessions sampled in all five provinces of Lazio were labeled ‘Spina’ with no or few variations. While some accessions actually had 100% genetic similarity (e.g., ‘Spina 1 RM’ vs. ‘Spina Nerola RM’), others were incredibly differentiated (‘Spina 1 FR’ vs. ‘Spina 2 RM’, 20.8% dissimilarity). The nomenclature could be rethought based on these molecular data.

Finally, the pairwise genetic similarity analyses (**Supplementary Table 1**) made it possible to identify eight anonymous samples belonging to the germplasm. In particular, five samples collected in the Frosinone province and generically labeled ‘Pero 38’, ‘Pero 39’, ‘Pero 42’, ‘Pero 43’ and ‘Pero 50’ showed a molecular profile identical to that of ‘Zunnina RM’. In contrast, ‘Pero 35’, ‘Pero 36’ and ‘Pero 37’ were found to be genetically different from all the varieties analyzed in the present study and could be temporarily registered as independent varieties.

### 3.3 SSRs are also useful for ploidy detection

SSR peak screening demonstrated triallelic loci in 122 genotypes (out of 250 unique genotypes). Except for the CH04e03 locus (where three alleles were never detected), all the other loci showed three alleles in at least 17 genotypes out of 250. CH01f07a was the locus with the highest number of individuals (52), showing three alleles.

As many as 71 genotypes showed only one locus with a third allele, 17 and 12 genotypes showed two and three loci, respectively, and 22 genotypes showed three peaks in a number of loci ranging from four to seven. Considering all genotypes that showed three alleles in at least one locus, we had a coverage of 49% putative triploids, the highest percentage found in any pear collection. However, similar results were described by Ferradini et al. (2017): of a total of 95 genotypes analyzed with nine SSR markers, 45% were triploids. Since somatic mutations generating chimerical or mosaic states or duplication events of a chromosome fragment might give rise to nonreal alleles, a single three-allelic locus is not always proof of triploid status (dos Santos et al., 2011; Ferradini et al., 2017). The percentage of triploids calculated only by relying on genotypes with two or more loci with a third allele dropped from 49% to 20% (69 genotypes out of 250). These results were in agreement with studies that followed the same consideration, displaying 20%, (Ferradini et al., 2017), 23.2% (Bielsa et al., 2021) and 27% (dos Santos et al., 2011) of triploids. All the ‘Angina’ accessions in the dataset showed a three-peak pattern, and nine out of ten accessions of ‘Bottiglia’ were triploids from the SSR marker results. In contrast, ‘De lu prete’ accessions from all the provinces of the Lazio region never showed a third peak. In all the other cases, no clear correlation between ploidy level and variety or geographical origin was detected.

### 3.4 Flow cytometry analysis confirmed the ploidy level of the pear germplasm

**Figure 1:**
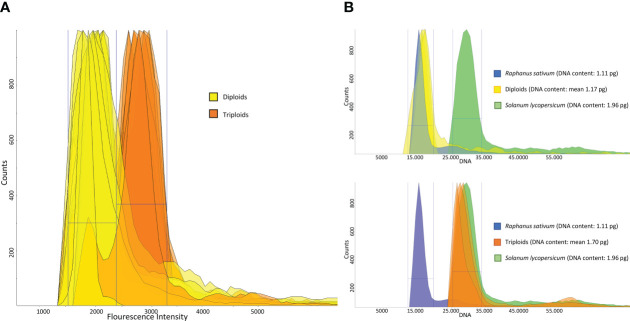
**(A)** DAPI-based flow cytometry measurements of 50 pear accessions. The peaks in yellow indicate the diploid region, whereas the orange-colored peaks represent the triploid area. **(B)** Genome size estimates of three diploid and three triploid accessions. Each peak represents the total DNA fluorescence emission of propidium iodide (PI)-stained leaf nuclei of diploids (yellow, upper part) and triploids (orange, bottom part). In both cases, each sample was cochopped and corun with two reference samples, namely, *Raphanus sativum* L. (blue peak) and *Solanum lycopersicum* L. (green peak).

To confirm the presence of triploid genotypes (estimated through SSR analysis), flow cytometry analyses were conducted. Among the 50 samples analyzed, we confirmed the results obtained from the SSR analysis for 48 samples (**Figure 1A**). ‘Cocozzola’ (Frosinone), which never showed a three-peak pattern from genotyping, was triploid according to cytometric measurements, while ‘Uaousa Renato’ (Frosinone), which showed the presence of three alleles for one locus, was diploid. This latter finding partially confirmed the hypothesis that a single three-allelic locus is not always proof of triploid status (dos Santos et al., 2011; Ferradini et al., 2017).

Since slight differences were observed among the flow cytometry (DAPI) measurements of samples characterized by the same ploidy level, three diploid and three triploid accessions were further investigated for their DNA content.

From the few data available in the Plant DNA C-value Database, (Royal Botanical Garden Kew Plant, 2022), the diploid *Pyrus communis* is characterized by an average 2C value of 1.18 pg, while no information is available for triploids. Similarly, Niu et al. (Niu et al., 2020) reported that the 2C value of all diploid pear species tested (*Pyrus bretschneideri* Rehd., *Pyrus pyrifolia* Nakai., *Pyrus ussuriensis* Maxim., *Pyrus communis* L., *Pyrus betulifolia*, *Pyrus sinkiangensis Yü.*), was always 1.11 ± 0.21 pg. The same authors reported that three local varieties of Xinjiang (China) were triploids, with an average 2C value of 1.52 pg.

For the purpose of the analysis, the nuclei of three diploid and three triploid accessions were costained along with those extracted from *Raphanus sativus* L. (2C = 1.11 pg) and *Solanum lycopersicum* L. (2C = 1.96 pg). Reference standards were chosen because the sizes of their genomes were comparable with the known predicted sizes of diploid and triploid accessions of pear. The diploid accessions ‘Invernale FR’, ‘Spina FR’ and ‘Di Carpello RM’ showed 2C values of 1.19 pg, 1.18 pg and 1.14 pg, respectively (mean = 1.17 pg, **Figure 1B**), in line with the data available in the literature. In contrast, the genome sizes of the triploid accessions ‘Coccozzola FR’, ‘Sammontana FR’ and ‘Bottiglia RM’ were 1.75 pg, 1.73 pg and 1.62 pg, respectively (mean = 1.70 pg, **Figure 1B**), which were slightly higher than those reported by Niu et al. (Niu et al., 2020).

From all cytometric data obtained, we could say that the SSR markers were efficient as good discriminants of the triploid genotypes analyzed, although cytometric analysis remains a pivotal tool for precisely measuring the level of ploidy. Interestingly, other than diploidy and triploidy, we did not detect any other level of ploidy. Results from many studies have shown no evidence of natural tetraploids observed in *Pyrus communis*, and the occurrence of so many triploids could be explained only by a lack of gamete reduction during meiosis, as documented by Sehic et al. (2012) and Baccichet et al. (2020). The most accepted theory is therefore that triploids in pear originate through the fertilization of an unreduced diploid egg cell with a haploid pollen cell. The high percentage of triploids is usually observed in collections of local varieties, which are selected and propagated by farmers, since they are appreciated for their phenotype, and consist of fruits that are larger and from higher trees than diploids (Capucci, 1940; Ferradini et al., 2017; Baccichet et al., 2020). Confirming the presence of triploids could be useful not only for traceability purposes but also when planning crosses in breeding programs, as triploids are difficult to use due to their disparate gamete formation and putative sterility.

### 3.5 Genetic diversity within the collection and population structure analysis

**Figure 2:**
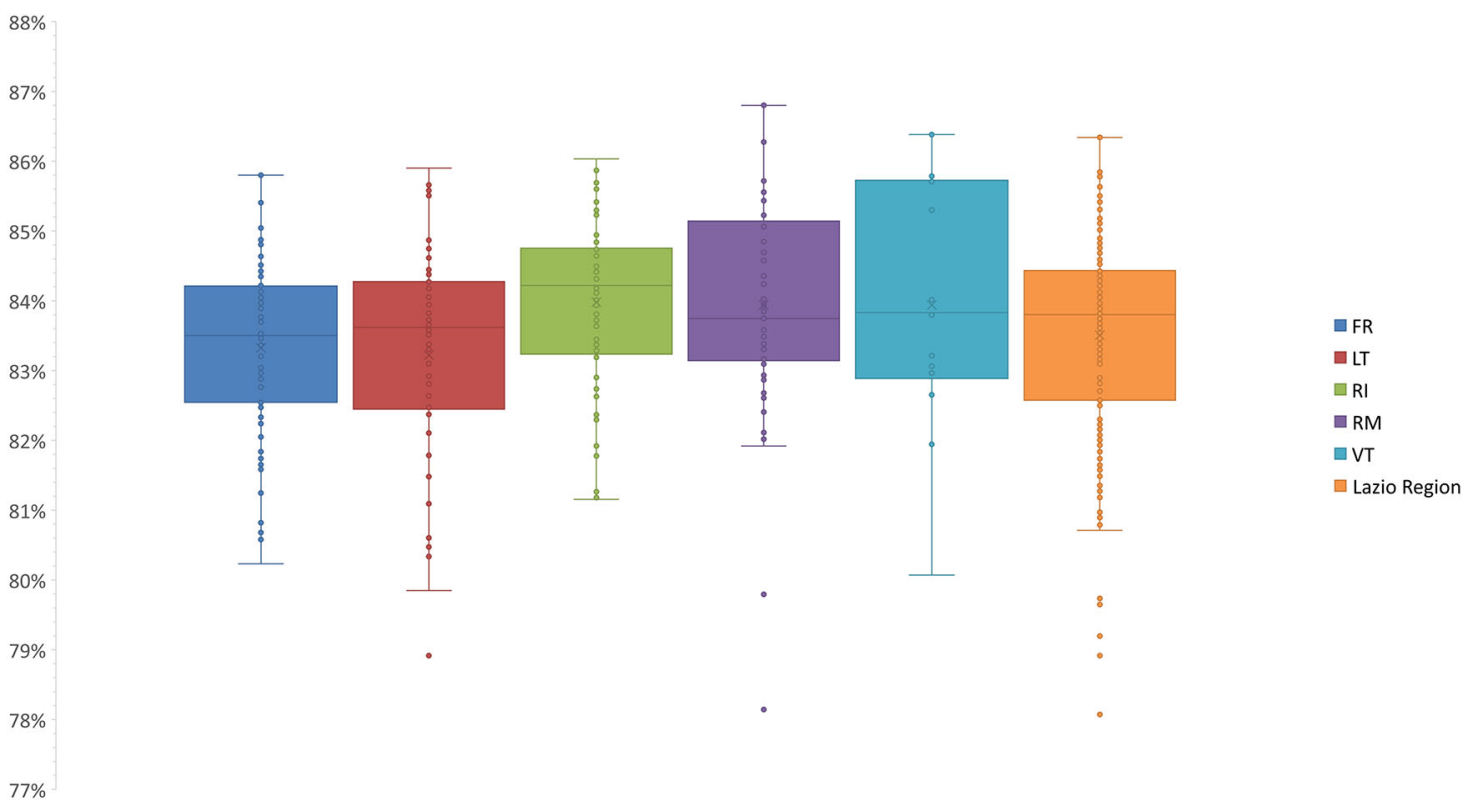
Statistics of the median genetic similarity (MGS) within the varieties of five provinces. The second and third quartiles are marked inside the rectangle and are divided by a bold bar (median). Dots outside show the outlier varieties, and each color represents the respective province of the Lazio Region (Frosinone, FR; Latina, LT; Roma, RM; Rieti, RI; Viterbo, VT).

The pairwise comparisons-based genetic similarity matrix (**Supplementary Table 1**) was also useful to investigate the overall genetic diversity of the pear germplasm. The genetic similarity values ranged from 71% to 100%. Those samples sharing 100% similarity have already been discussed as synonymy cases (**Supplementary Table 2**). The lowest similarity values were often observed between varieties differing in both place of origin and phenotype. For instance, one of the lowest similarity values (71%) was observed between ‘Brutta e Bona VT’, characterized by small brown fruits and originally from the Sardinia region, and ‘Spina 1 FR’, characterized instead by large and green fruits and originally from Capri (Campania region). Based on the genetic similarity matrix, the median values among the varieties of each province were calculated (**Figure 2** **)**.

Notably, we could not detect any correlation between genetic similarity and geographic origin, since no significant differences were observed in comparison with the median values calculated among the varieties as a whole (**Figure 2**). This finding was also confirmed by the analysis of molecular variance (AMOVA): only 1.7% of the variance was found among provinces, while 98% of the variance was found within provinces, which agrees with many outbreeding species (Jiang et al., 2009; Miranda et al., 2010; Wolko et al., 2015; Wuyun et al., 2015; Ferradini et al., 2017; Baccichet et al., 2020).

**Figure 3:**
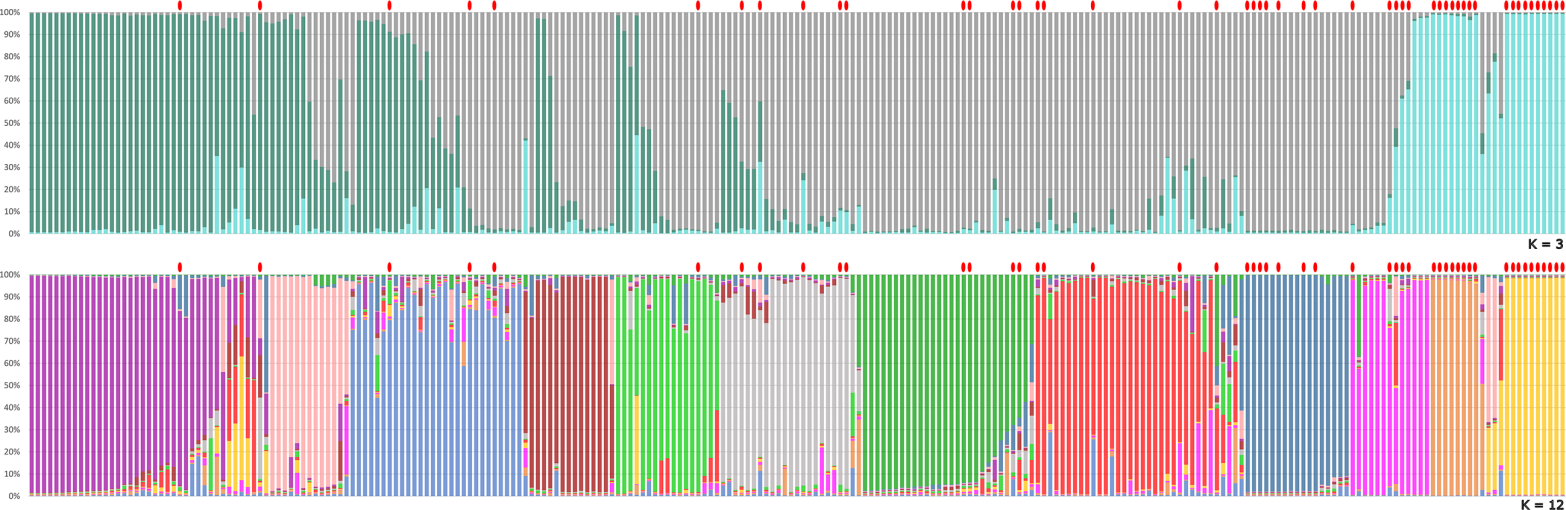
Genetic structure of the pear germplasm collection as estimated by STRUCTURE using the SSR marker dataset. Each sample is represented by a vertical-colored histogram partitioned into K = 3 and K = 12 representing the estimated membership. The proportion of ancestry (%) is reported on the abscissa axis. Red dots indicate triploid accessions with two or more loci with a third allele.

**Figure 4:**
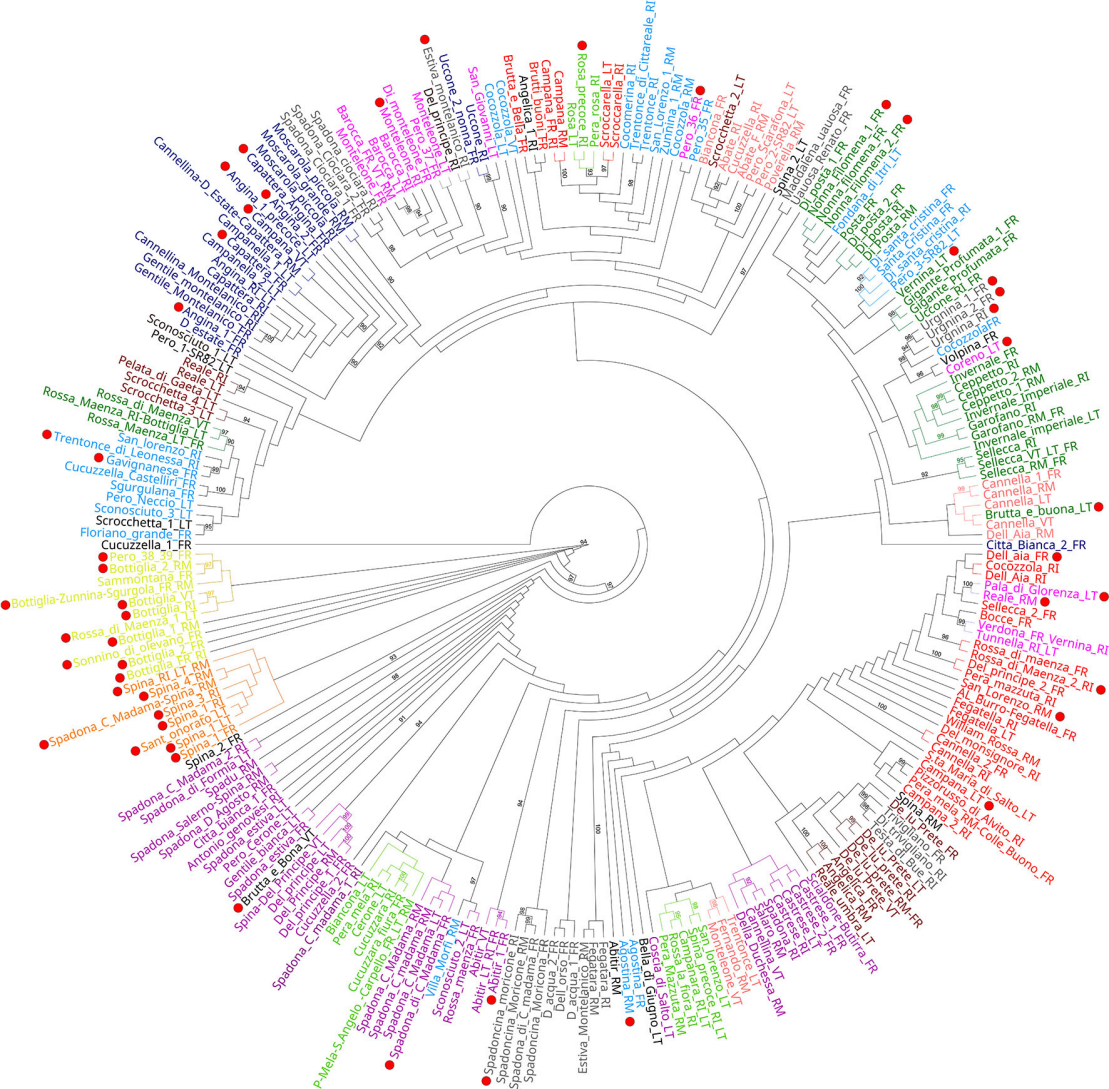
Maximum likelihood dendrogram topology representing the genetic relationships among the different genotypes. Bootstrap values (≥ 90) are highlighted to support the branches on the dendrogram, and red dots indicate accessions with two or more loci with a third allele.

The genetic structure analysis of the 250 unique genotypes, developed following the procedure described by Evanno et al. (Evanno et al., 2005), showed the maximum ΔK values at K = 3 and K = 12 (**Figure 3**). From K = 12, the division into 12 clusters displayed a clear vision about the possible common ancestors shared among varieties. Many admixed patterns were also observed, probably because of hypothetical hybridization events. These results were further integrated with an ML-based dendrogram that largely supported the outcome found through the ancestry analysis (**Figure 4** **)**. In fact, most of the clusters observed from STRUCTURE analysis also matched with specific groupings detected within the ML dendrogram, even if some exceptions were observed. Most of the accessions labeled ‘Spina’ were clustered in the orange cluster (**Figure 3**, lower panel and **Figure 4**) and resulted in all triploids. An analogous situation was observed for the yellow cluster: all the accessions were triploids and prevalently represented by samples labeled ‘Bottiglia’, an ancient French variety. The yellow and orange groups, in addition to being the only ones entirely composed of triploids, were the most genetically distant from the rest of the germplasm (**Figure 4**). These two clusters likely share a common ancestor that is different from the rest of the germplasm, as suggested by the population structure findings obtained for K = 3 (**Figure 3**, upper part). In fact, the accessions belonging to these two groups were the only ones showing full membership to a third ancestor (turquoise) not shared by any of the remaining samples of the germplasm.

The last group showing a considerable number of triploid accessions was the dark-blue cluster. Here, ‘Angina’, ‘Campanella’, ‘Capattera’ (all synonym names of the same variety), ‘Moscarola’ and ‘Cannellina’ were grouped together.

The distinctiveness of the yellow, orange and dark-blue triploid clusters was also confirmed by principal coordinate analysis (**Supplementary Figure 1**), where dimension-1 explained 37.3% of the cumulative molecular variation, and dimension-2 explained 24.2% of the cumulative molecular variation. In the PCoA, the three triploid clusters were labeled by circles with the same colors used in the STRUCTURE analysis (**Figure 3**) and the dendrogram (**Figure 4**). Beyond this, no other correlation between ploidy and ancestry clusters was observed, since triploids were dispersed in all clusters. This would confirm the hypothesis according to which triploidization events (due to the lack of gamete reduction) would be frequent and independent of the origin or variety.

One last noteworthy cluster, partially detached from the rest of the germplasm (**Figure 4**) and gathering most of the accessions labeled ‘Spadona’, was the purple cluster. From the few historical data available (Capucci, 1940; Bianco et al., 2014; Baccichet et al., 2020), ‘Bottiglia’ and ‘Spadona’ are closely related varieties, while the origin of ‘Spina’ is not clear, even if some sources claim it to be an ancient variety from Capri. However, many synonyms are used for ‘Spina’, ‘Spadona’ and ‘Bottiglia’ varieties, leading to the hypothesis of a possible correlation among the three groups of varieties. Furthermore, the allochthonous origins of these varieties might explain their genetic divergence from the rest of the local varieties.

## 4 Conclusions

In the last 20 years, the Lazio Region has been at the forefront in the protection of local varieties through the establishment, maintenance and continuous updating of regional biodiversity repositories. The recent choice to integrate the use of molecular tools to safeguard local varieties from the risk of genetic erosion represents a further step forward. Molecular markers enable us to determine with greater precision the diversity existing in a germplasm, highlighting cases of homonymy and synonymy. In fact, beyond the protection of local varieties, one of the goals is also to avoid redundancy in the collections to reduce their management costs. In the specific case of pear, molecular analyses also revealed novel genetic insights into the genetic structure and ploidy level of the germplasm. The molecular profiles produced for each accession are being used to integrate the information already available in the regional catalogs (https://www.arsial.it/biodiversita/registro-volontario-regionale/). Harmonizing the marker sets used for germplasm characterization would be of paramount importance to ensure the use of these profiles at the national and international levels.

## Data availability statement

The original contributions presented in the study are included in the article/**Supplementary Material**, further inquiries can be directed to the corresponding author/s.

## Author contributions

FaP and GB: conceptualization. SD and FaP: methodology. SD: formal analysis. SD: data analysis. SD and FaP: writing—original draft preparation. SD, FaP, IM, FrP, and GB: writing—review and editing. GB and FaP: supervision and project administration. GB and IM: funding acquisition. All authors have read and agreed to the published version of the manuscript.
