# Supplementary material for: Management of genetic erosion: The (successful) case study of the pear (Pyrus communis L.) germplasm of the Lazio region (Italy)
Source: Front Plant Sci. 2023 Jan 9;13:1099420. doi: 10.3389/fpls.2022.1099420 (PMC9868429; doi:10.3389/fpls.2022.1099420)
Supplement: Supplementary file 1 [file DataSheet_1.pdf]

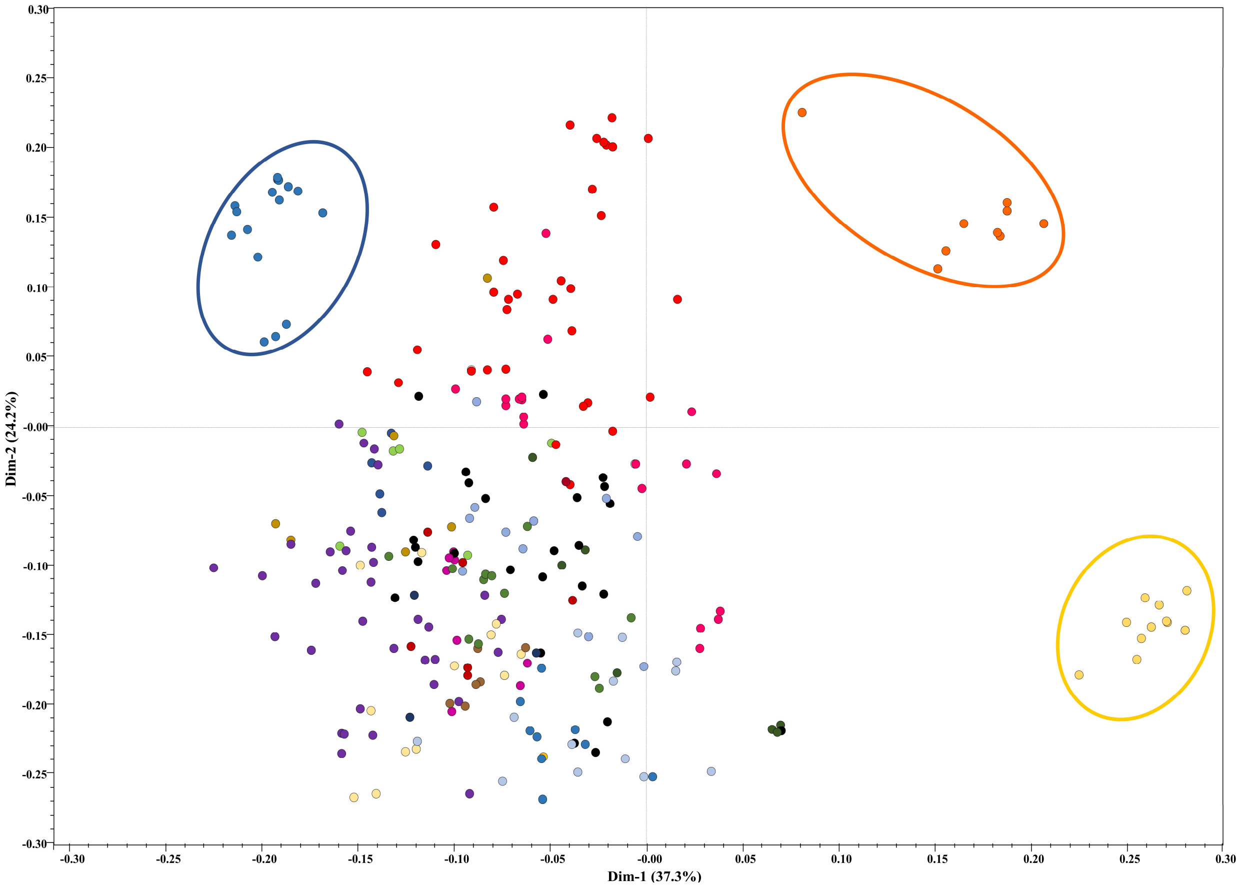

Supplementary Figure 1. Principal coordinate analysis of the genetic variation based on microsatellite markers for *Pyrus communis* L., with 37.3% and 24.2% of genetic variation explained by Axes 1 and 2, respectively.
